# Did School Food and Nutrient-Based Standards in England Impact on 11–12Y Olds Nutrient Intake at Lunchtime and in Total Diet? Repeat Cross-Sectional Study

**DOI:** 10.1371/journal.pone.0112648

**Published:** 2014-11-19

**Authors:** Suzanne Spence, Jennifer Delve, Elaine Stamp, John N. S. Matthews, Martin White, Ashley J. Adamson

**Affiliations:** 1 Institute of Health and Society, Newcastle University, Newcastle upon Tyne, England; 2 Human Nutrition Research Centre, Newcastle University, Newcastle upon Tyne, England; 3 School of Mathematics and Statistics, Newcastle University, Newcastle upon Tyne, England; 4 Fuse, UKCRC Centre for Translational Research in Public Health, Newcastle upon Tyne, England; University of Alabama at Birmingham, United States of America

## Abstract

**Introduction:**

In September 2009, middle and secondary schools in England were required to comply with food and nutrient-based standards for school food. We examined the impact of this policy change on children’s lunchtime and total dietary intake.

**Methods:**

We undertook repeat cross-sectional surveys in six Northumberland middle schools in 1999–2000 and 2009–10. Dietary data were collected from 11–12 y olds (*n* = 298 in 1999–2000; *n* = 215 in 2009–10). Children completed two consecutive 3-day food diaries, each followed by an interview. Linear mixed effect models examined the effect of year, lunch type and level of socio-economic deprivation on children’s mean total dietary intake.

**Results:**

We found both before and after the introduction of the food and nutrient-based standards children consuming a school lunch, had a lower per cent energy from saturated fat (−0.5%; p = 0.02), and a lower intake of sodium (−143 mg; p = 0.02), and calcium (−81 mg; p = 0.001) in their total diet, compared with children consuming a home-packed lunch. We found no evidence that lunch type was associated with mean energy, or absolute amounts of NSP, vitamin C and iron intake. There was marginal evidence of an association between lunch type and per cent energy NMES (p = 0.06). In 1999–2000, children consuming a school lunch had a higher per cent energy from fat in their total diet compared with children consuming a home-packed lunch (2.8%), whereas by 2009–10, they had slightly less (−0.2%) (year by lunch type interaction p<0.001; change in mean differences −3%).

**Conclusions:**

We found limited evidence of an impact of the school food and nutrient-based standards on total diet among 11–12 year olds. Such policies may need to be supported by additional measures, including guidance on individual food choice, and the development of wider supportive environments in school and beyond the school gates.

## Introduction

Reducing childhood overweight and obesity are public health priorities [1]; improving diet is central to achieving a healthier lifestyle and losing weight [2], [3]. Although there is some evidence of a levelling off in childhood obesity [4], [5], in 2011–12, the National Child Measurement Programme in England identified a third of 10–11 y olds as overweight or obese [6], and socio-economic disparities persist [4], [7].

Obesity has been found to track from adolescence to adulthood [8], [9]; one potentially contributing factor is poor dietary patterns [9]. The English National Diet and Nutrition Survey found per cent energy from saturated fat and non-milk extrinsic sugar (NMES) exceeded the Dietary Reference Value of 11%; per cent energy from NMES was highest in 11–18 y olds (15.3%) [10]. Only 11% of boys and 8% of girls met the recommended ‘5-a-day’ for fruit and vegetables [10]. Certain micronutrients, for example iron, were below the Reference Nutrient Intake.

Improving dietary intake in this age group is complex. During adolescence there is increasing independence in food choice [11] with social factors playing a crucial role[12]–[14]. For adolescents, food and drink consumption is related to ‘identity’ and ‘status’ [12], [13]. One effort to tackle adolescent’s diets has been a change in government policy requiring middle and secondary schools in England to comply with food and nutrient-based standards for school food from September 2009 [15]. These specify the provision of certain foods and the average nutrient content school lunches must provide over a three week menu cycle [16]. The majority of studies exploring the impact of the food and nutrient-based standards have focused on change in lunchtime intake in primary schools[17]–[24]; few have reported on middle and secondary schools [25], [26]. Following the implementation of nutritional standards, Fletcher et al. reported the increased selling of junk food by students and suggested these standards ignore the wider contextual issues associated with food choice [14]. Studies have also highlighted negative aspects of school lunches, for example pricing [14] and a preference to socialise with friends at lunchtime [12]. Findings also reveal negative aspects of the dining environment, for example overcrowding, queuing [12], [14], [27] and noise [14].

With limited findings from quantitative studies, it is important to examine whether the food and nutrient-based standards could potentially affect nutrient intake among adolescents. In this paper we report research which examined the impact of the introduction of food and nutrient-based standards for school lunch on the lunchtime and total diet of a representative sample of children aged 11–12 years, between 1999–2000 (before) and 2009–10 (after) introduction of the policy in England.

## Methods

### Ethics statement

Ethical approval was granted by Newcastle University ethics committee (reference 000011/2007). In 2009–10, Newcastle University ethics committee granted approval for opt-out to be used as the method of consent (reference 00011/2009). Parents were provided with a written information letter about the study and a consent form, however, they were only required to return the consent form if they did not wish their child to participate. Newcastle University ethics committee approved our study design, methods and the consent procedure used for this study. All the data in this study were anonymised.

### Study design, setting and participants

Cross-sectional studies were undertaken in middle schools in Morpeth, Ashington and Newbiggin-by-the-Sea in Northumberland, North East England over two academic years: 1999–2000 (before) and 2009–10 (after implementation of the standards). These areas were previously selected to be representative of schools with catchment populations across the socio-economic spectrum [28], [29]. The 1999–2000 data were collected as part of a series of studies conducted in Northumberland[11], [30]–[32] to track changes in dietary patterns and used as the baseline in this study. The same schools were invited by letter in 2009 to participate in this study. This was followed up with a school visit to answer questions and ascertain interest. During discussions with heads of schools they suggested consent should be changed from ‘opt-in’ (as used in the previous studies in these schools) to ‘opt-out’. The rationale was that by using opt-in we excluded children whose parents failed to return forms sent by schools, rather than just those children whose parents actively did not want their child to participate. After obtaining documented support from heads and school governors, an amendment to the Newcastle University Ethics approval was granted for the use of opt-out in 2009–10 (reference 00011/2009). One head preferred that his school continued to use opt-in (this was the smallest school) and the decision was taken to retain this school despite a different method used in the consent process. Children could still exclude themselves by not completing food diaries and were free to leave the study at any time.

All children in year 7 were eligible to participate. A presentation was given at individual schools and each child received a parental information letter and a consent form to return if they did not wish to participate. Participating children received a unique identification number to anonymise data. All data were stored securely according to Newcastle University policies and regulations.

### Data

#### Dietary consumption

We used dietary assessment methods identical to those used in the previous Northumberland studies [11], [30]. This method has been described in detail [11], [27], [30], [33] and validated [29], [34]; a brief overview is provided here. Verbal instructions on how to complete the diary were given to each participating child; the diary also included an example page with instructions. Children recorded the day, date and time when food or drink was consumed, descriptions of items and amounts of foods/drinks for two consecutive three-day periods (for example Thursday, Friday, Saturday and Sunday, Monday, Tuesday). On the fourth day the child was interviewed by a trained researcher to clarify information recorded and estimate portion size using food models and a photographic food atlas for 11–14 y olds [35]. Foods were coded using McCance and Widdowson’s Integrated Composition of Food dataset [36]. If available, school recipes were used to code school lunch, and if not, foods were coded as above. Foods were categorised into ‘school lunch’, ‘home-packed lunch’ and ‘food consumed outside of school hours’. In common with the large majority of secondary schools in England [37]none of the schools permitted pupils to leave school premises at lunchtime. The macro- and micronutrients examined in this paper relevant to the nutrient-based standards are: energy (kcals), per cent energy from fat, saturated fat, and non-milk-extrinsic sugars (NMES); and absolute amounts of non-starch polysaccharides (NSP) (g), sodium (mg), vitamin C (mg), calcium (mg) and iron (mg).

#### Socio-economic status

Socio-economic status was estimated using the English Index of Multiple Deprivation (IMD) 2007 [38], allocated using individual children’s postcodes. IMD is calculated at lower layer super output areas in England and provides a single deprivation score based on seven domains: income, employment, health and disability, education, skills and training, barriers to housing and services, crime and living environment [38]. The IMD scores were categorised into quintiles for the analyses: quintile 1 included children living in the 20% least deprived areas, quintile 5 included children living in the 20% most deprived areas.

### Main outcome measures

Main outcome measures were mean daily intakes of macro- and micronutrients in ‘school lunch’, ‘home-packed lunch’ and total diet, measured as indicated below.

### Statistical analysis

We undertook three sets of analyses. The first considered the change in school lunch take-up. A linear model was fitted directly to the proportions taking school lunch using maximum likelihood (fitted in R using optim), which allowed for differences between IMD quintiles, between years and their interaction. The second examined the change at lunchtime in children’s mean macro- and micronutrient intake from a school or home-packed lunch on school days only between 1999–2000 and 2009–10. The third analysis considered the intake of macro- and micronutrients in children’s total diet: this explored the effect of year (before and after the food and nutrient-based standards), lunch type (school or home-packed lunch) and level of deprivation. We used linear mixed effect models to examine the effect of these variables; interactions between variables were considered (year by lunch type, year by level of deprivation and lunch type by level of deprivation). Where there was no evidence for a particular interaction for a given nutrient, the interaction was excluded from the final model. All analyses adjusted for the effect of gender and day type (week or weekend day). Within each model random effects were included for school and child. Data were analysed using Stata version 11 and models were fitted using *xtmixed*. Vitamin C was log transformed for analysis, and for this variable geometric means and ratios are reported in tables.

## Results

### Study sample characteristics

Table 1 shows the number of children who consented to take part by year and reasons for exclusion. There was a similar percentage of males and females participating in 1999–2000 (m = 47%; f = 53%) and 2009–10 (m = 50%; f = 50%), and there was no evidence of a statistically significant difference in children’s mean IMD score (p = 0.3).

**Table 1:** Number of children consenting and reasons for exclusion in 1999–2000 and 2009–10.

	1999–2000	2009–10
Number consenting	*n* = 424	*n* = 295
Reasons for exclusion:		
From non-comparable school*	19	–
Mixed lunch†	96	73
No postcode	6	7
Completed less than 6 food diary days	5	0
Number included in analysis	298	215

*Non-comparable school: one school had closed from 1999–2000 to 2009–10.

† Mixed lunch means a child having both a school and home-packed lunch.

From Table 2 it can be seen that school lunch take-up was similar across all IMD quintiles in 1999–2000: between 1999–2000 and 2009–10 there was a decrease in the percentage of children consuming a school lunch, with evidence that the decrease differed across the IMD quintiles. The fall in school lunch take-up decreased linearly across the IMD quintiles (linear by year interaction p = 0.01, likelihood ratio test), with a fall of 61 percentage points in the least deprived group compared with a mean reduction of 32 percentage points in the most deprived group.

**Table 2:** Number (percentage) of children consuming a school lunch by year and level of deprivation.

	1999–2000			2009–10			[1999–00]-[2009–10]
Level of deprivation	No. having school lunch	Total *	(%)	No. having school lunch	Total	(%)	Decrease in %
Quintile 1 (least)	44	54	(81)	12	55	(20)	61
Quintile 2	43	55	(78)	11	41	(27)	51
Quintile 3	40	50	(80)	15	34	(44)	36
Quintile 4	38	49	(78)	10	26	(38)	40
Quintile 5 (most)	75	90	(83)	30	59	(51)	32
All children	240	298	(81)	78	215	(36)	45

*Total = no. having school and home-packed lunch.

### Lunchtime diet

Tables 3 and 4 show the change in children’s mean daily nutrient intake in school and home-packed lunches respectively between 1999–2000 and 2009–10, compared with the nutrient-based standards [16]. In school lunches, between 1999–2000 and 2009–10, there was strong evidence of a decrease in mean energy intake (mean difference −232 kcals; p<0.001), per cent energy from fat (−9.9%; p<0.001) and saturated fat (−1.9%; p<0.001), and in absolute amounts of sodium (−390 mg; p<0.001), but also a decrease in mean NSP (−0.7 g; p<0.001) and iron intake (−0.7 mg; p<0.001). We found no evidence of a change in per cent energy from NMES (1.1%; p = 0.2), mean vitamin C (ratio 1.0; p = 0.7) and marginal evidence of a change in calcium intake (−22.3 mg; p = 0.05) (Table 3). In 1999–2000, children’s mean energy and sodium intake from school lunch were above the target for the current school nutrient-based standards. By 2009–10, mean intakes were below these targets [16]. In 1999–2000, mean intakes of NSP, calcium, iron and vitamin C intake were below the nutrient-based standards [16]; these deficits persisted in 2009–10 (Table 3).

**Table 3:** Lunchtime: Change in children’s mean daily nutrient intake from school lunch between 1999–2000 and 2009–10, and nutrient-based standards [16].

Nutrient	Standard	Consumption from school lunch				
		1999–2000	2009–10	[2009–10]–[1999–2000]		
		n = 240	n = 78			
		mean*		mean difference	95% CI for difference	p-value†
Energy (kcals)	610	729	497	−232	−276; −189	<0.001
% energy fat	–	40.6	30.7	−9.9	−11.4; −8.6	<0.001
% energy saturated fat	–	12.5	10.6	−1.9	−2.7; −1.3	<0.001
% energy NMES	–	11.9	13.0	1.1	−0.4; 2.7	0.2
NSP (g)	min 4.9	3.9	3.2	−0.7	−1.0; −0.4	<0.001
Sodium (mg)	max 714	908	518	−390	−453; −328	<0.001
Vitamin C (mg) ‡	min 12.3	28.8	28.2	1.0	0.9; 1.1	0.7
Calcium (mg)	min 350	206.5	184.2	−22.3	−44.4; −0.3	0.05
Iron (mg)	min 5.2	2.8	2.1	−0.7	−0.9; −0.5	<0.001

*Mean adjusted for gender.

† P-value derived from a linear mixed effects model.

‡ Vitamin C log transformed; geometric means and ratios reported.

**Table 4:** Lunchtime: Change in children’s mean daily nutrient intake in home-packed lunch between 1999–2000 and 2009–10, and nutrient-based standards [16].

Nutrient	Standard	Consumption from home-packed lunch				
		1999–2000	2009–10	[2009–10]–[1999–2000]		
		n = 58	n = 137			
		mean*		mean difference	95% CI for difference	p-value†
Energy (kcals)	610	605	578	−27	−77; 23	0.3
% energy fat	–	34.0	32.3	−1.7	−4.0; 0.7	0.2
% energy saturated fat	–	14.1	14.2	0.1	−1.3; 1.5	0.8
% energy NMES	–	17.8	17.1	−0.7	−3.0; 1.7	0.6
NSP (g)	min 4.9	2.9	3.4	0.5	0.04; 1.0	0.03
Sodium (mg)	max 714	954	889	−65	−165; 34	0.2
Vitamin C (mg) **^‡^** ^§^	min 12.3	26.9	34.7	1.3	1.1; 1.6	0.006
Calcium (mg)	min 350	223.2	292.1	68.9	21.1; 116.7	0.005
Iron (mg)	min 5.2	2.6	2.4	−0.2	−0.5; 0.1	0.3

*Mean adjusted for gender.

† P-value derived from a linear mixed effects model.

‡ Vitamin C log transformed; geometric means and ratios reported.

In packed lunches, between 1999–2000 and 2009–10, there was a statistically significant increase in absolute amounts of mean NSP (mean difference 0.5 g; p = 0.03), calcium (68.9 mg; p = 0.005) and vitamin C intake (1.3; p = 0.006) (Table 4). We found no evidence of a change in mean energy (−27 kcals; p = 0.3), per cent energy from fat (−1.7%; p = 0.2), saturated fat (0.1%; p = 0.8), NMES (−0.7%; p = 0.6), or absolute amounts of sodium (−65 mg; p = 0.2) or iron intake (−0.2 mg; p = 0.3) (Table 4).

### Total diet

**Figure 1:**
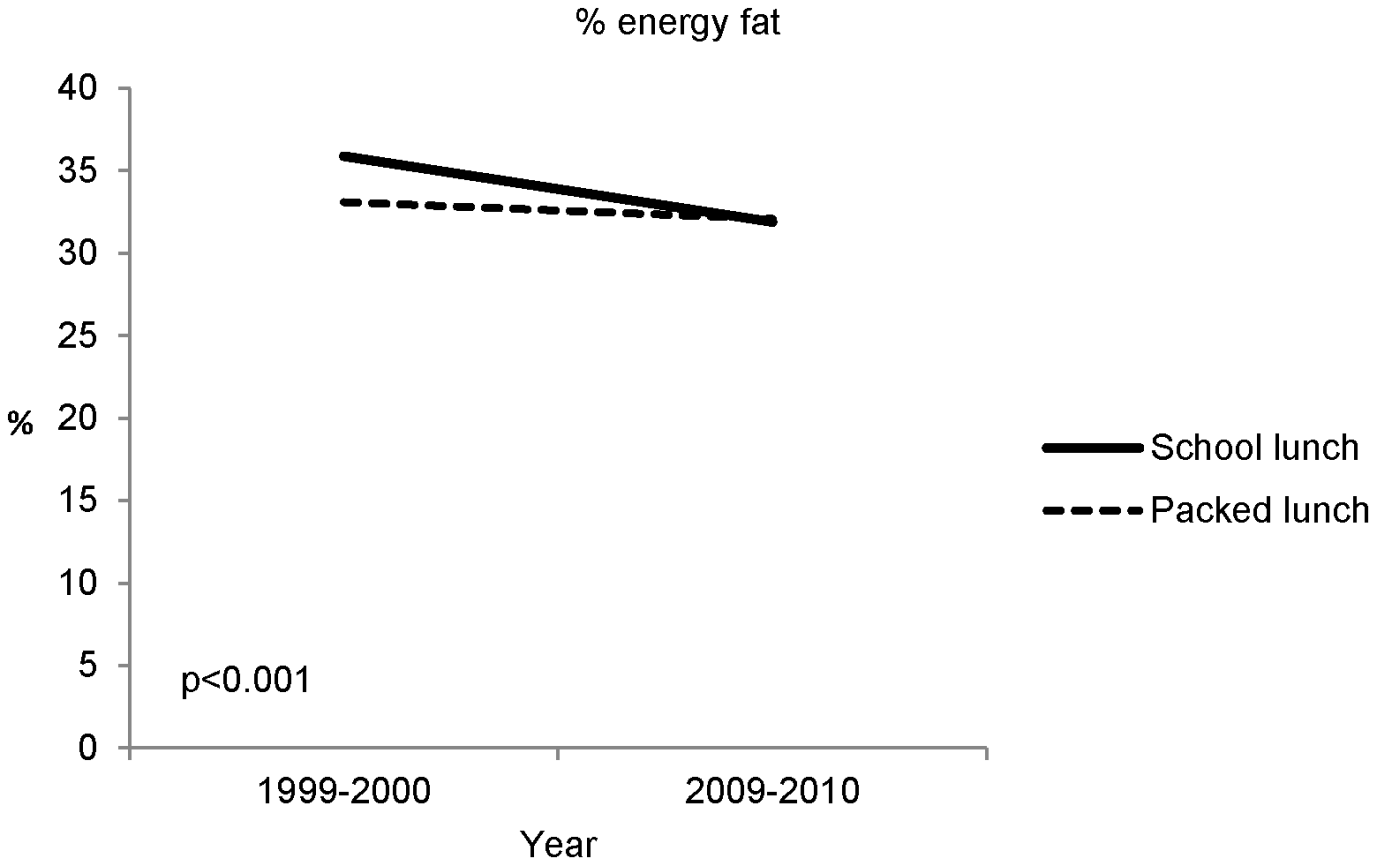
Total diet: The effect of year and lunch type interaction on children’s per cent energy from fat (adjusted for gender, level of deprivation and day type).

The results from the total diet analysis are shown in Tables 5, 6, 7 and Figure 1. Table 5 shows the effect of year (before and after the food and nutrient-based standards), Table 6 the effect of lunch type (school or home-packed lunch) and Table 7 the effect of level of deprivation. There was evidence of a year by lunch type interaction only for per cent energy from fat (Figure 1).

**Table 5:** Total diet: The effect of year on children’s mean daily nutrient intake and Dietary Reference Values/Reference Nutrient Intakes (DRV/RNI) [39].

Nutrient	DRV/RNI	1999–2000*2009–10	[2009–10]–[1999–2000]		
		Mean†		Mean difference	95% CI for difference	p-value‡
Energy (kcals)	M§ = 2220; F§ = 1845	1924	1665	−259	−332; −185	<0.001
% energy saturated fat	≤11	12.9	12.7	−0.2	−0.6; 0.2	0.4
% energy NMES	≤11	16.5	16.0	−0.5	−1.3; 0.4	0.3
NSP (g)	–	10.8	9.9	−0.9	−1.5; −0.3	0.002
Sodium (mg)	1600	2593	2118	−475	−590; −361	<0.001
Vitamin C (mg)∥	35	67.6	79.4	1.2	1.1; 1.3	<0.001
Calcium (mg)	M = 1000; F = 800	698	802	104	60; 149	<0.001
Iron (mg)	M = 11.3; F = 14.8	9.6	8.6	−1.0	−1.6; −0.5	<0.001

*Number of children participating in 1999–2000 (*n* = 298) and 2009–10 (*n* = 215).

† Mean adjusted for gender, day-type, lunch type and level of deprivation.

‡ 95% CI and p-value derived from a linear mixed effects model.

§ M (male) F (female).

∥ Vitamin C log transformed; geometric means and ratios reported.

**Table 6:** Total diet: The effect of lunch type (school or home-packed lunch) on children’s mean daily nutrient intake and Dietary Reference Values/Reference Nutrient Intakes (DRV/RNI) [39].

Nutrient	DRV/RNI	Packed (PL)*School (SL)		[SL-PL]		
		Mean†		Mean difference	95% CI for difference	p-value‡
Energy (kcals)	M§ = 2220; F§ = 1845	1792	1788	−4	−78; 71	0.9
% energy saturated fat	≤11	13.2	12.7	−0.5	−0.9; −0.1	0.02
% energy NMES	≤11	16.9	16.0	−0.9	−1.8; 0.0	0.06
NSP (g)	–	10.1	10.2	0.1	−0.5; 0.7	0.8
Sodium (mg)	1600	2490	2347	−143	−261; −26	0.02
Vitamin C (mg) ∥	35	70.8	72.4	1.0	0.9; 1.1	0.5
Calcium (mg)	M = 1000; F = 800	778	697	−81	−127; −35	0.001
Iron (mg)	M = 11.3; F = 14.8	9.2	8.8	−0.4	−0.9; 0.2	0.2

*Number of children participating in 1999–2000 (*n* = 298) and 2009–10 (*n* = 215).

† Mean adjusted for year, gender, day-type and level of deprivation.

‡ 95% CI and p-value derived from a linear mixed effects model.

§ M (male) F (female).

∥ Vitamin C log transformed; geometric means and ratios reported.

**Table 7:** Total diet: The effect of level of deprivation on children’s mean daily nutrient intake and Dietary Reference Values/Reference Nutrient Intakes (DRV/RNI) [39].

Nutrient	DRV/RNI	Level of deprivation					
		1 (least deprived) *	2	3	4	5 (most deprived)	
		Mean†					p-value‡
Energy (kcals)	M§ = 2220; F§ = 1845	1773	1830	1813	1821	1747	0.4
% energy fat	≤35	33.5	33.6	33.9	34.1	34.4	0.2
% energy saturated fat	≤11	12.6	12.9	12.9	13.1	12.8	0.7
% energy NMES	≤11	16.3	16.7	16.2	15.8	16.4	0.8
NSP (g)	–	10.1	10.4	10.4	10.5	9.8	0.5
Sodium (mg)	1600	2421	2452	2407	2484	2310	0.2
Vitamin C (mg) ∥	35	81.3	79.4	77.6	67.6	61.7	<0.001
Calcium (mg)	M = 1000; F = 800	744	763	753	723	680	0.04
Iron (mg)	M = 11.3; F = 14.8	8.9	9.4	9.4	8.8	8.5	0.08

*Number of children participating in 1999–2000 (*n* = 298) and 2009–10 (*n* = 215).

† Mean adjusted for year, lunch type, gender and day-type.

‡ P-value derived from a linear mixed effects model.

§ M (male) F (female).

∥ Vitamin C log transformed; geometric means and ratios reported.

In total diet, between 1999–2000 and 2009–10, there was strong evidence of a decrease in mean energy intake (mean difference −259 kcals; p<0.001), and absolute amounts of sodium (−475 mg; p<0.001), but also a decrease in NSP (−0.9 g; p = 0.002), and iron intake (−1.0 mg; p<0.0001). Mean calcium and vitamin C intake increased (104 mg; p<0.001 and ratio 1.2; p<0.001 respectively) (Table 5). We found no evidence of a change in per cent energy from saturated fat (−0.2%; p = 0.4) or NMES (−0.5%; p = 0.3) (Table 5). In 2009–10, children’s per cent energy from saturated fat and NMES remained above the recommendation of ≤11% [39]. Mean vitamin C intake was the only micronutrient to meet the Reference Nutrient Intake [39].

Table 6 shows the effect of lunch type (school or home-packed lunch) on children’s mean total dietary intake, with data from before and after the introduction of the legislation combined. There was clear evidence that children who consumed a school lunch both before and after the implementation of the food and nutrient-based standards had a lower per cent energy from saturated fat (mean difference −0.5%; p = 0.02), and absolute amounts of sodium (−143 mg; p = 0.02), and calcium intake (−81 mg; p = 0.001) compared with children who consumed a packed lunch (Table 6). We found no evidence of a statistically significant effect of lunch type on mean energy, or absolute amounts of NSP, vitamin C and iron intake in total diet. We found marginal evidence of an effect on per cent energy from NMES (−0.9%; p = 0.06) (Table 6).

In both 1999–2000 and 2009–10, we found strong evidence of a level of deprivation effect on mean vitamin C intake. Mean intakes were lowest for children in the most deprived quintile (test for the effect of level of deprivation: p<0.001, Table 7). We found no evidence of an effect on mean energy, per cent energy from fat, saturated fat, NMES, or absolute amounts of NSP and sodium intake. We found marginal evidence of an effect on mean calcium and iron intake. Mean intakes were lowest for those in the most deprived quintile (test for the effect of level of deprivation: p = 0.04 and p = 0.08 respectively) (Table 7).

For one nutrient, per cent energy from fat, we found a statistically significant year by lunch type interaction on children’s total dietary intake (p<0.001; Figure 1). This was because there was a markedly higher per cent energy from fat in school lunches compared with packed lunches in 1999–2000 (35.9% and 33.1% respectively; mean difference 2.8%), whereas the corresponding difference in 2009–2010 was very small (31.9% and 32.1% respectively; −0.2%). The change in these differences: (2009/10–1999/00) is (−0.2) −2.8 = −3% (95% CI −4.4 to −1.4; see Figure 1). We found no evidence of any statistically significant year by level of deprivation or lunch type by level of deprivation interactions.

## Discussion

### Summary of key findings

Between 1999–2000 and 2009–10, the number of children consuming a school lunch decreased with the greatest decline in children from more affluent families. At lunchtime, in 2009–10, we found that children eating school lunches consumed a healthier diet with regard to per cent energy from fat, saturated fat, NMES and sodium, but had a lower mean micronutrient intake than children consuming packed lunches. In total diet, between 1999–2000 and 2009–10, there was a statistically significant decrease in mean intakes of energy and sodium, but also a decrease in NSP and iron, while vitamin C and calcium intake increased. We found no evidence of a change in per cent energy from NMES or saturated fat. There was limited evidence that a child’s lunch type was associated with a change in children’s mean total dietary intake. The only association found between year (before and after the introduction of the food and nutrient-based standards) and a child’s lunch type (school or home-packed lunch) was in relation to per cent energy from fat consumed. By 2009–10, children who consumed a school lunch had a slightly lower intake of per cent energy from fat in their total diet compared with those who consumed a home-packed lunch. We found little evidence that mean nutrient intakes were associated with level of deprivation.

### Relationship to other studies

In 2009–10, school lunch take-up in the six Northumberland middle schools participating in this study was 36%. A study in English academies and city technology colleges found school lunch take-up was 37.6% in 2010–11 [40].

There is limited research examining the impact (before and after implementation) of the food and nutrient-based standards in England on dietary intake at lunchtime and the impact of this policy change on total diet in 11–12 y olds. A number of studies have examined nutritional intake in this age group at school or in their total diet. What this study adds is a consideration of school and home-packed lunch both separately and in the context of total diet, prior to and following a major change in school food policy.

At lunchtime, we found mean energy, NSP, calcium and iron intakes were below the nutrient-based standards in both school and home-packed lunches; however, vitamin C was above. These findings are similar to those from a national survey of 80 secondary schools in England [26]. In school lunch, per cent energy from fat, saturated fat and NMES were comparable with the national survey. In home-packed lunch, we found a lower per cent energy from fat, but a higher per cent energy from saturated fat and NMES compared with the national survey. In contrast to other studies, [26], [41], [42] we found that a school lunch provided a lower mean energy, NSP, and micronutrient intake than a home-packed lunch. Our findings concur with those by Hur et al [43] and Taylor et al [44] who found children who consumed a school lunch had a lower mean energy intake than children consuming a home-packed lunch. Similarly Taylor et al [44] also found lower intakes of some micronutrients, such as iron and vitamin C. The lower mean intakes of micronutrients for children consuming a school lunch in our study may be due to the lower mean energy intake which highlights the need for increased nutrient quality with lower energy intakes. These findings show some inconsistencies in energy and some micronutrient intakes in studies that have investigated what children eat in a school or home-packed lunch. These differences may be due to a number of factors, for example: age of children studied and variation in food provision and wider support to which children are exposed, however, differences due to dietary data collection methods cannot be excluded. A study by Pearce et al [45] showed that some portion sizes of foods on offer had decreased since the implementation of the policy; variation in portion sizes served across schools may also explain inconsistencies in findings.

A study by Fung et al [46] that examined change in children’s total diet pre to post-school lunch policy in Canada (Grade 5 children) reported similar findings to our study. For example, they found a decrease in per cent energy from fat and absolute amounts of sodium; and also a decrease in mean fibre intake. In contrast to our study they found mean iron intake increased. [46] In total diet, we found children’s mean energy, calcium and iron intake were below recommended intakes [39]; per cent energy from saturated fat and NMES, and absolute amounts of sodium were above. This is similar to findings from 11–18 y olds in the National Dietary and Nutrition Survey (NDNS) [47]. Between 1999–2000 and 2009–10, we found a decrease in energy, per cent energy from fat and saturated fat, and little change in per cent energy from NMES. Mean vitamin C and calcium intake increased, but iron decreased; these findings are also similar to the trends observed in the NDNS [47], [48]. This decrease in mean energy and per cent energy from fat was also observed in a previous study in Northumberland examining the macronutrient intake in 11–12 y olds between 1980 and 2000 [11]. In contrast, in this later study we found no evidence of a change in per cent energy from NMES which remained above recommended intakes [38] (16% compared with 11%). This suggests products with a high sugar content, such as breakfast cereals, confectionery and fruit juices, remain a constant element of children’s dietary intake.

### Strengths and Limitations

This is the first study in a middle school setting to use a natural experimental, repeat cross-sectional design before and after the implementation of the standards to evaluate the impact both at lunchtime and in total diet [49]. A limitation of this approach is attributing causality [24]. National implementation of the food and nutrient-based standards in primary, middle and secondary schools prevented the use of a stronger study design with a control group and prospective follow-up of individual children [24]. This study was limited to the North East of England, so, findings may not be generalisable [24]. Socio-economic status was estimated using IMD, which does not measure individual levels of deprivation, and is therefore subject to potential misclassification bias [50]. We used identical prospective dietary data collection methods at both time points to ensure consistency. The data collection method relied on self-report and was potentially subject to misreporting [51]. We collected two three-day periods of dietary data to limit this bias.

### Conclusions and implications

The school environment offers an opportunity to influence dietary intake. Yet, our findings have shown limited evidence of the food and nutrient-based standards affecting total diet in this age group, which is in contrast to the results among younger children [24]. Reasons for this may be a reduction in the proportion of children consuming a school lunch, less than full compliance with the food and nutrient-based standards, or individual food choice. School lunches have potential to improve children’s dietary intake but only if they are consumed. This study found a decrease in school lunch take-up which suggests the importance of addressing the wider social aspects of overcrowding, noise and queues in school dining rooms [12], [14], [27] to provide an attractive environment conducive to healthy eating. Other factors may also be associated with a decrease in school lunch take-up. The standards limit the frequency of serving of certain foods and also restrict what food and drink can be served. A process evaluation undertaken parallel to this study highlighted that parents of younger children (4–7 y olds) supported the restriction of food choice. However, there was more ‘*ambivalence* in the parents of middle school children (11–12 y olds) for who personal preference was an important issue. In the 11–12 y olds some parents were more concerned about value for money and that children had enough to eat, therefore, some parents preferred to give their children a home-packed lunch as this was considered cheaper and ‘*less risky’*. [27] This may be reflected in the lower decline of take-up in children from more deprived families who would be more likely to be in receipt of free school meals.

We noted variation in provision between schools and not all of the middle schools that participated in this study were fully compliant with the standards. For policy changes to be implemented effectively in schools and achieve the potential impact, support needs to be available for all stakeholders, including catering suppliers, head teachers and school catering staff. Policies affecting the provision of school food should also take account of the views of students using these facilities, [12], [14] both at policy development and implementation stages. Strategies to support and guide food choice by pupils remains important; on a positive note children consuming school lunches were shown to eat a lower per cent energy from fat, saturated fat, NMES and sodium than those consuming home-packed lunches, but fewer micronutrients, which is a cause for concern. This study shows improvements are needed in the nutritional content of both school lunches and home-packed lunches. Our findings highlight a persistent need to improve dietary intake in this age group both at school and throughout the day. Across the socio-economic spectrum, children’s consumption of saturated fat and NMES remain above the recommended limits, while micronutrients remain below. In 1984, Hackett et al. noted the need for a focus on nutrient density in children’s diets due to falling energy intakes [33]. This remains relevant today. These findings reiterate the importance of considering the influence of the wider environment in this age group, and also, the need for both policy and societal approaches.
